# Anti-*Toxoplasma gondii* effect of tylosin in vitro and in vivo

**DOI:** 10.1186/s13071-024-06157-0

**Published:** 2024-02-10

**Authors:** Ru-Xia Han, Pi-Cheng Jiang, Bing Han, Huai-Yu Zhou, Yong-Liang Wang, Jing-Yu Guan, Zhi-Rong Liu, Shen-Yi He, Chun-Xue Zhou

**Affiliations:** 1 Department of Pathogen Biology, School of Basic Medical Sciences, Cheeloo College of Medicine, Shandong University, Jinan, 250012 Shandong Province People’s Republic of China; 2 Shandong Public Health Clinical Center, Cheeloo College of Medicine, Shandong University, Jinan, 250012 Shandong Province People’s Republic of China

**Keywords:** *Toxoplasma gondii*, Tachyzoite, Tylosin, Sulfadiazine sodium

## Abstract

**Background:**

*Toxoplasma gondii* is an important protozoan pathogen with medical and veterinary importance worldwide. Drugs currently used for treatment of toxoplasmosis are less effective and sometimes cause serious side effects. There is an urgent need for the development of more effective drugs with relatively low toxicity.

**Methods:**

The effect of tylosin on the viability of host cells was measured using CCK8 assays. To assess the inhibition of tylosin on *T. gondii* proliferation, a real-time PCR targeting the B1 gene was developed for *T. gondii* detection and quantification. Total RNA was extracted from parasites treated with tylosin and then subjected to transcriptome analysis by RNA sequencing (RNA-seq). Finally, murine infection models of toxoplasmosis were used to evaluate the protective efficacy of tylosin against *T. gondii* virulent RH strain or avirulent ME49 strain.

**Results:**

We found that tylosin displayed low host toxicity, and its 50% inhibitory concentration was 175.3 μM. Tylsoin also inhibited intracellular *T. gondii* tachyzoite proliferation, with a 50% effective concentration of 9.759 μM. Transcriptome analysis showed that tylosin remarkably perturbed the gene expression of *T. gondii*, and genes involved in “ribosome biogenesis (GO:0042254)” and “ribosome (GO:0005840)” were significantly dys-regulated. In a murine model, tylosin treatment alone (100 mg/kg, i.p.) or in combination with sulfadiazine sodium (200 mg/kg, i.g.) significantly prolonged the survival time and raised the survival rate of animals infected with *T. gondii* virulent RH or avirulent ME49 strain. Meanwhile, treatment with tylosin significantly decreased the parasite burdens in multiple organs and decreased the spleen index of mice with acute toxoplasmosis.

**Conclusions:**

Our findings suggest that tylosin exhibited potency against *T. gondii* both in vitro and in vivo, which offers promise for treatment of human toxoplasmosis.

**Graphical Abstract:**

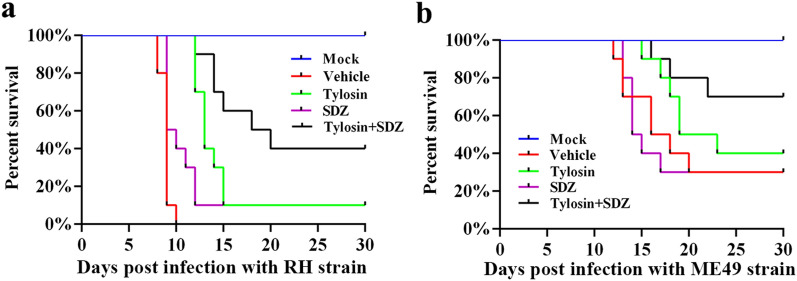

**Supplementary Information:**

The online version contains supplementary material available at 10.1186/s13071-024-06157-0.

## Background

*Toxoplasma gondii* is an obligate intracellular protozoan parasite that can infect almost all warm-blooded animals, including humans [1]. Approximately 20% to 30% of the world’s human population carries this parasite [2]. Acquired infection with *T. gondii* in healthy persons is generally an asymptomatic or a flu-like infection. However, in immunocompromised individuals and in the developing fetus, *T. gondii* multiplies greatly and results in life-threatening diseases, such as central nervous system diseases, retinochoroiditis and pneumonitis [3, 4]. Studies also show that latent toxoplasmosis has been implicated in the risk for behavioral changes, including depression, schizophrenia and bipolar disorder [5]. Unfortunately, to date, no commercial vaccine for human use has been developed to prevent infection or no chemotherapeutic agents to eradicate this parasite from infected individuals.

Currently, the most effective available therapeutic combination is pyrimethamine plus sulfadiazine. They impair the parasite replication and survival through the inhibition of folic acid synthesis [6]. Although they are active on tachyzoites, the therapy is not able to eliminate bradyzoites from the organism. Besides, they can cause severe hematological side effects and embryopathies [7]. Other alternative therapies, such as atovaquone, clindamycin and spiramycin, have been widely used [8]. Although they can cure *T. gondii* infection effectively, most toxoplasmosis patients will relapse without long-term therapy. In addition, the above commonly used anti-*Toxoplasma* drugs are highly effective against the tachyzoite form of the parasite but do not show significant activity against the latent tissue cysts that make *Toxoplasma* a long-term chronic infection. Therefore, the exploration for new alternative therapeutic options against *T. gondii* infection is vital and a prime concern.

Tylosin is a macrolide antibiotic, which has a broad spectrum of activity against mycoplasmas and gram-positive aerobic bacteria [9]. It is approved for therapy of a variety of livestock and poultry infectious diseases, such as mastitis, respiratory diseases and dysentery in farm animals [10]. Like other macrolide antibiotics, tylosin exerts its antimicrobial action by binding to the 50S ribosome subunit and inhibiting protein synthesis [11]. In this study, we evaluated the anti-*T. gondii* potential of tylosin by using experimental mouse models infected with *T. gondii* virulent RH strain or avirulent ME49 strain. Our findings indicate that tylosin exhibits potency against *T. gondii* both in vitro and in vivo, suggesting that it may be a promising agent to treat human toxoplasmosis.

## Methods

### Animals and parasites

Female KM mice with ages ranging from 2 to 3 months were purchased from the Laboratory Animal Center of Shandong University, China. All mice were placed in an environment with appropriate temperature and ventilation, 12 h of light and 12 h of darkness. Mice were given sterilized food and water ad libitum. Mice were acclimated for 1 week before being used in the experiment.

Tachyzoites of *T. gondii* RH and ME49 strains were cultivated in confluent monolayers of human foreskin fibroblasts (HFF) (HS27; ATCC: CRL-1634) in Dulbecco's Modified Eagle Medium (DMEM) with 10% fetal bovine serum (FBS, Gibco), 2 mM glutamine, 100 U/ml penicillin and 10 µg/ml streptomycin at 37 °C, 5% CO_2_.

### Chemicals

Tylosin was obtained from GlpBio (CAS: 1401-69-0, USA). A stock solution (100 mM) was prepared in 100% dimethyl sulfoxide (DMSO), kept at -80 ℃ and aliquoted to desirable concentrations when needed. Sulfadiazine sodium was purchased from Sigma-Aldrich (CAS: 547-32-0, Germany).

### In vitro cytotoxicity study

HFF cells were cultured in 96-well plates (Corning Inc., USA) at a density of 1 × 10^4^ cells per well for 12 h. Then, cells were incubated in fresh complete medium containing tylosin at different concentrations (1 μM, 10 μM, 50 μM, 100 μM, 200 μM, 300 μM, 400 μM and 500 μM) for 48 h. As previously described, cell viability was assessed using the Cell Counting Kit-8 (CCK-8) (Beyotime Biotechnology, China) [12]. Ten μl CCK-8 solution was added to each well, and the cells were incubated at 37 °C for an additional 2 h. The absorbance values of each well were monitored at 450 nm on an ELx800 microplate reader (BioTek Inc., USA).

### Parasite quantification

Parasite burden was measured by an absolute quantitative PCR method. Parasite replication was assessed by extracting genomic DNA (gDNA) from samples, followed by measuring the amplification of the *T. gondii* B1 gene with parasite-specific primers (5'-TGAGTATCTGTGCAACTTTGG-3' and 5'-TCTCTGTGTACCTCTTCTCG-3'). A standard curve was generated with plasmids containing B1 gene. A StepOne real-time PCR machine with FastKing One Step RT- PCR MasterMix (Tiangen, China) was used to determine parasite proliferation in each sample relative to a standard curve of plasmid DNA templates.

### In vitro anti-*Toxoplasma gondii* activity of tylosin

Free RH tachyzoites were pre-treated with tylosin (50 μM) or vehicle (0.1% DMSO) for 3 h, 6 h, 9 h or 12 h at 4 °C. Next, HFF cells were infected with pre-treated parasites at MOI = 1 for 60 h. Parasite replication was measured by an absolute quantitative PCR method as described above.

### Determination of EC_50_

Purified RH-GFP tachyzoites at 10^5^/well were added to 24-well plates, where HFF cells were seeded (2 × 10^5^ cells/well) and cultured for 12 h. After 2 h, the extracellular parasites were washed away using PBS. Tylosin at final concentrations of 2.5, 5, 10, 20, 30, 40 or 50 μM was added. Medium was used as a negative control. After 48 h of incubation, images were acquired with a Zeiss Axio Vert.A1 microscope (Carl Zeiss AG, Germany) with a 40 × objective using ZEN imaging software (Carl Zeiss). The fluorescence intensity of RH-GFP was measured using Image J software. Ten randomly selected fields were counted for each triplicate sample. EC_50_ on *T. gondii* was determined as the sample concentration for which 50% of parasite growth was inhibited.

### Inhibition assay of tylosin on intracellular *Toxoplasma*

HFF cells were cultured on six-well plates (5 × 10^5^ cells/well/2 ml) for 12 h at 37 °C and 5% CO_2_. The cells were infected with tachyzoites of *T. gondii* RH strain at MOI = 1. After 2 h, free parasites were removed, and the cells were treated with tylosin (50 μM) or 0.1% DMSO (vehicle) for 48 h or 72 h. Intracellular parasites and attached cells were collected, and genomic DNA was isolated. Intracellular parasites were then quantified by an absolute quantitative PCR method.

### Immunofluorescent assays (IFAs)

HFF cells were seeded on coverslips in 24-well plates (2 × 10^5^ cells/well) for 12 h at 37 °C and 5% CO_2_. The cells were infected with tachyzoites of *T. gondii* RH strain at MOI = 1. After 2 h, extracellular parasites were washed away using PBS, and the cells were treated with tylosin (50 μM) or 0.1% DMSO (vehicle) for 48 h. Cells were fixed with 4% paraformaldehyde and permeabilized with 0.1% Triton X-100 for 15 min, incubated with mouse anti-TgIMC8 antibody (1:200 dilution) for 20 min, stained with DAPI (Thermo Fisher Scientific) for 5 min and incubated with Alexa Fluor 488-conjugated goat anti-mouse lgG secondary antibodies (Life Technologies, USA) for 20 min. Images were acquired with a Zeiss Axio Vert.A1 microscope (Carl Zeiss AG, Germany) with a 40 × objective using ZEN imaging software (Carl Zeiss).

### RNA extraction

Free tachyzoites (approximately 10^7^ parasites) were treated with tylosin (50 μM) or 0.1% DMSO (vehicle) for 6 h at 4 °C. Parasites were collected and immediately snap-frozen in liquid nitrogen and stored at – 80 °C until further use. Total RNA was extracted using TRIzol reagent as previously described [13]. RNA quality was determined using an Agilent 2100 bioanalyzer (Agilent Technologies, Santa Clara, CA).

### mRNA sequencing

Transcriptome sequencing was performed by the BGI Sequencing Company (Shenzhen, China) with DNBSEQ™ sequencing technology platforms. Total RNA was fragmented and enriched using oligo (dT) magnetic beads, followed by cDNA synthesis. Sequencing libraries were generated after the removal of ribosomal RNA. Libraries were sequenced using DNBSEQ-500 platform, averagely generating > 6 Gb bases per sample. Low quality reads and reads with adaptors or with unknown bases (N bases > 5%) were filtered to get the clean reads. After filtering, the clean reads with high quality were aligned to the *T. gondii* reference genome (https://www.ncbi.nlm.nih.gov/genome/30?genome_assembly_id = 899143) using HISAT2 (v2.0.4) [14, 15]. Finally, clean reads were normalized to FPKM (fragments per kilobase of exon model per million mapped fragments) as relative gene expression levels. Differential gene expression analysis was performed using the DESeq2 Bioconductor package (v3.16), and the Benjamini-Hochberg multiple correction test was applied to control false discovery rate (FDR). Differentially expressed (DE) genes with an adjusted *P* value cutoff of 0.05 were considered statistically significant for further analysis.

Gene ontology (GO) enrichment analysis of DE mRNAs was done using web-based GO software (https://geneontology.org/) [16]. GO terms with a corrected *P* value (*q* value) < 0.05 were considered significantly enriched. Pathway enrichment analysis was performed by using a web-based Kyoto Encyclopedia of Genes and Genomes database (KEGG, https://www.genome.jp/kegg/) [17].

### in vivo anti-*Toxoplasma gondii* activity of tylosin

To assess the protective efficacy of tylosin against acute infection, mice in infection groups were administered intraperitoneally (i.p.) with 100 RH strain tachyzoites or 200 ME49 strain tachyzoites in sterile PBS and isolated by syringe lysis and filtration of an infected HFF monolayer. Meanwhile, a negative control group was mock infected with sterile PBS alone. Treatment was initiated 24 h post infection, and infected mice were assigned to administration with vehicle (10% DMSO, 40% PEG-300, 5% Tween 80, and ddH2O, i.p.), tylosin (100 mg/kg in 10% DMSO, 40% PEG-300, 5% Tween 80, and ddH2O, i.p.), SDZ (200 mg/kg in ddH2O, i.g.) or tylosin plus SDZ. The drugs were administrated once a day, and the treatment lasted for 7 days. Mice were observed at 7 a.m., noon and 7 p.m., and percent survival was recorded at each time point for 30 days.

### Parasite quantification in mouse tissues

Parasite quantification in tissues was performed following the methods by Santoro et al. [18]. Animal tissues were collected at 8 days post infection and stored in individual vials at − 20 °C before genomic DNA extraction. For each sample, 1 g of tissue was individually homogenized, and 200 µl of the homogenate was used to extract genomic DNA according to the manufacturer’s protocols (DP304, Tiangen, China). A standard curve was obtained by linear regression analysis of the threshold cycle (Ct) value (y-axis) versus the log of the initial copy number present in each sample dilution (x-axis). PCR efficiency (E) was calculated as E = 10 (1/slope) ^−1^.

### Histological analysis

The spleen samples were collected at 8 days post infection and fixed in 10% neutral formaldehyde solution for 48 h. The fixed tissue was embedded in paraffin and cut into sections of 3 μm thickness. The tissue sections were stained with Hematoxylin and Eosin Staining Kit (Beyotime, China) according to manufacturer’s instructions. The H&E stained samples were observed under a light microscope (Olympus Optical Co., Tokyo, Japan). To give a quantitative assessment of the splenomegaly, spleen indexes can be defined as: Spleen index = (Weight of experimental organ (mg))/(Weight of experimental animal (g)) [19].

### Statistical analysis

All statistical analyses were performed using GraphPad Prism 7.0 software (GraphPad). All results were expressed as mean ± standard deviation (SD). Statistical differences were calculated by Student’s *t* test (two-tailed, unpaired), one-way *ANOVA* or two-way *ANOVA*. *P* value < 0.05 indicated that there was a statistically significant difference.

## Results

### Tylosin inhibits *T. gondii *growth in vitro

**Fig. 1:**
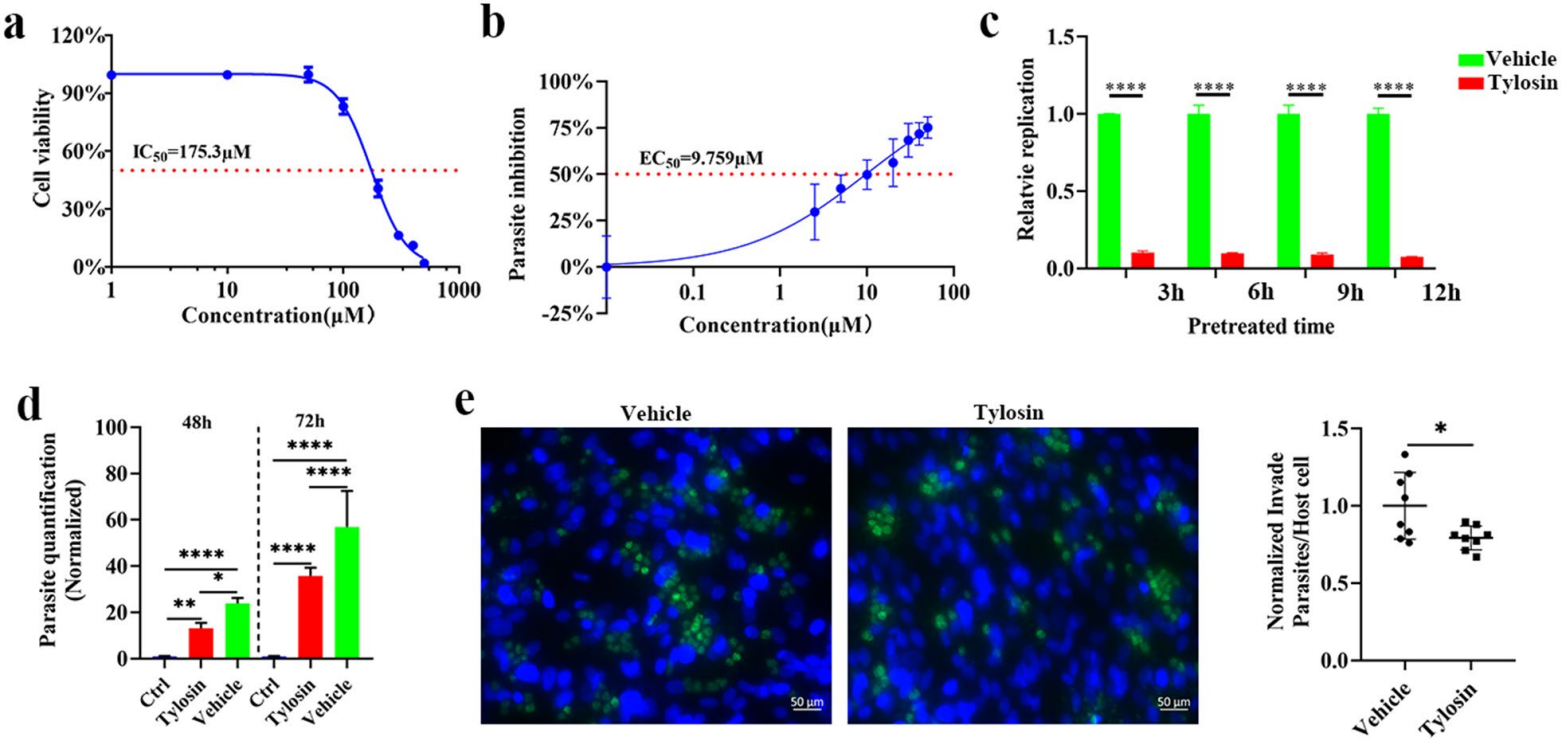
Effect of tylosin treatment on host cell viability and *Toxoplasma gondii* growth in vitro. **a** HFF cells were treated with tylosin at concentrations ranging from 1 to 500 μM for 48 h, and the IC_50_ value was obtained by CCK8 assays. **b** Inhibition of *T. gondii* type I parasites (RH-GFP) after tylosin treatment. The EC_50_ was determined. **c** Effects of tylosin (50 μM) on the activity of extracellular *T. gondii* RH tachyzoites. The *P* values by two-way ANOVA are indicated; ****P < 0.001. **d** Inhibitory effects of tylosin (50 μM) on the intracellular parasite (RH strain) replication. Samples in the control group (Ctrl) were collected at 2 h post infection, and other groups were collected at 48 h or 72 h post infection. The *P* values by one-way ANOVA are indicated; *P < 0.05, ** P < 0.01 and ***P < 0.005. **e** Inhibitory effects of tylosin on parasite infection. RH -infected HFFs were treated with tylosin (50 μM) or 0.1% DMSO (vehicle) for 48 h. The cells were stained with DAPI and mouse anti-IMC8 antibody. Representative immunofluorescence images of parasites treated with vehicle (0.1% DMSO) and tylosin are shown. Shown are the average values and standard deviations from one experiment representative of three independent experiments. The *P* values by Student’s *t*-test are indicated; *P < 0.05

A CCK-8 assay was performed to analyze the toxicity of tylosin on HFF cells in vitro. Cells were treated with different concentrations of tylosin for 48 h. As shown in Fig. 1a, when HFF cells were exposed to 50 μM, the cell proliferation rate was 99.6% (*P* > 0.05). When the cells were exposed to 100 μM tylosin, the proliferation rate fell to 83.11% (*P* < 0.001). The 50% inhibitory concentration (IC_50_) value was determined to be 175.3 μM (Fig. 1a). Therefore, the safe concentrations of tylosin for HFF cells were ≤ 50 μM. Tylosin was able to inhibit parasite growth, with an EC_50_ value of 9.759 μM on RH-GFP strain (Fig. 1b).

To better investigate the potential of anti-*Toxoplasma* activity of tylosin in vitro, we examined the proliferation rate of RH strain tachyzoites pretreated with tylosin (50 μM) or 0.1% DMSO (vehicle). As shown in Fig. 1c, tylosin significantly (*P* < 0.001) inhibited the activity of extracellular *T. gondii* tachyzoites in a time-dependent manner. Next, we determined the efficacy of tylosin against intracellular parasite growth. RH tachyzoites were allowed to invade host cells for 2 h. The extracellular parasites were removed, and fresh medium containing tylosin or 0.1% DMSO (vehicle) was added and incubated for 48 h or 72 h. As shown in Fig. 1d, tylosin significantly inhibited the proliferation of intracellular *T. gondii* tachyzoites (*P* < 0.005). Meanwhile, IFA results also confirmed the inhibitory effect of tylosin against *T. gondii* infection (Fig. 1e).

### Tylosin alters the transcriptome of *T. gondii*

**Fig. 2:**
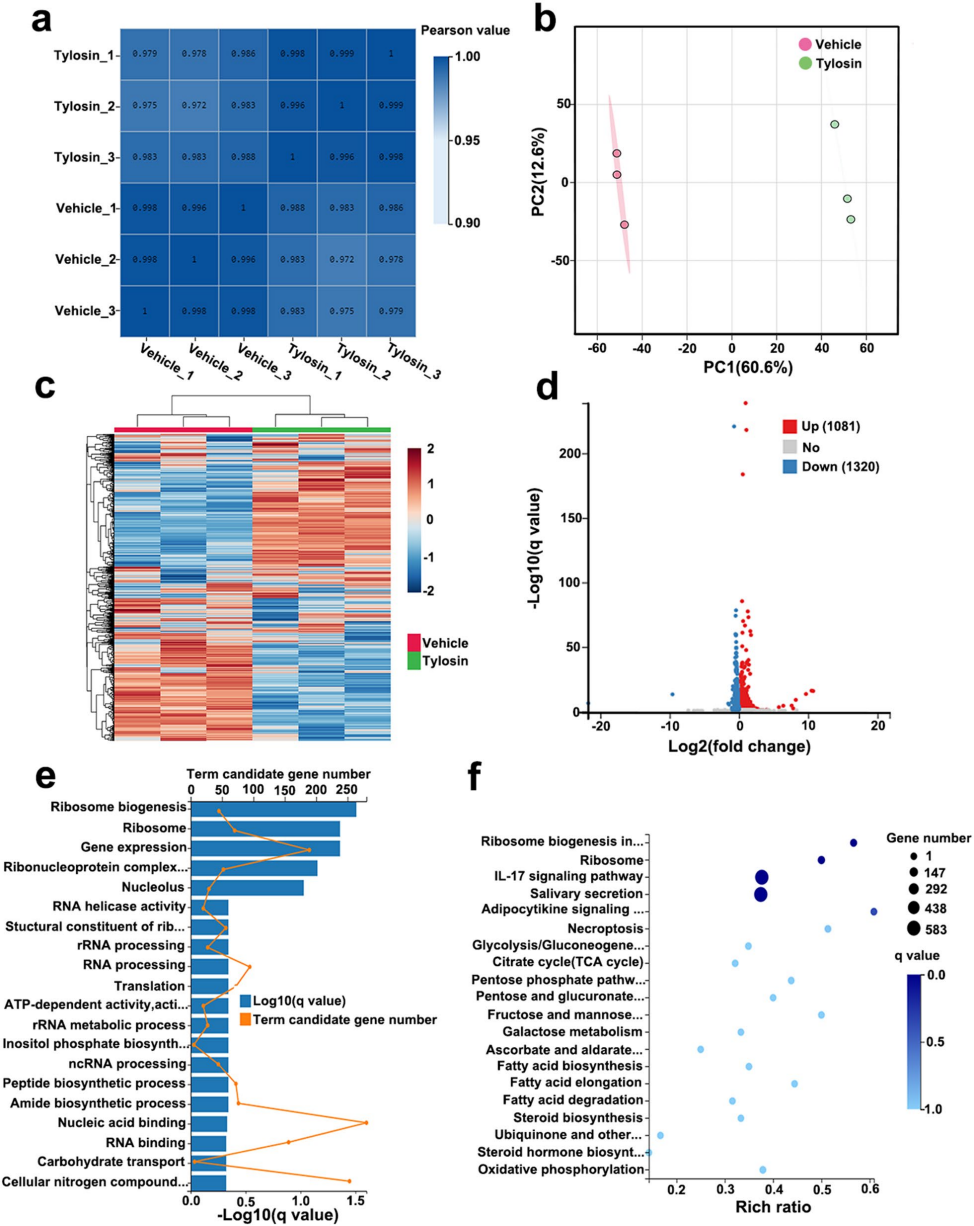
Incubation with tylosin alters the transcriptome of *Toxoplasma gondii* RH tachyzoites. **a** Heatmap for the Pearson correlation coefficient between samples. **b** Principal component analysis (PCA) score scatter plot of *Toxoplasma* samples based on FPKM values. **c** Unsupervised hierarchical clustering of R-seq data. FPKM value is normalized so that blue represents low expression and reddish color represents high expression. Columns were hierarchically clustered based on a complete linkage using Pearson correlation coefficient as the distance measure. **d** Volcano plot showing *q* values (–log10) versus RNA expression ratios between tylosin treatment group and vehicle group (0.1% DMSO). **e** Gene Ontology (GO) analysis of DE genes. The X-axis label denotes –log10(*q* value), whereas the Y-axis label represents the corresponding GO terms. In addition, the yellow dot indicates the number of DE genes. **f** The top 20 significantly enriched KEGG pathways of the DE genes. The X-axis label shows the rich ratio. The Y-axis label shows the KEGG pathway terms. The color of the dots represents log10 (*q* value), and the size of the dot represents the number of DE genes enriched in the KEGG pathway

To analyze the effect of tylosin on *T. gondii* transcriptome, RNA-Seq analysis was performed. *Toxoplasma gondii* RH tachyzoites were treated with 50 μM tylosin or 0.1% DMSO (vehicle) for 6 h. To investigate the RNA expression patterns, a heatmap for the Pearson correlation coefficient between samples was built. As shown in Fig. 2a, a Pearson correlation matrix clearly demonstrates the differences between vehicle group and the drug-treated group. Principal component analysis (PCA) score plot and unsupervised hierarchical clustering clearly differentiate tylosin-treated groups from vehicle group (Fig. 2b and c). Then, a volcano plot was used to analyze differentially expressed (DE) genes. As shown in Fig. 2d, 1081 upregulated and 1320 downregulated genes have been identified between tylosin-treated group and vehicle group. Detailed information of the DE genes is listed in Additional file 1: Table S1.

To better understand the roles of these DE genes, Gene Ontology (GO) enrichment analysis was performed. As shown in Fig. 2e, the top 20 most enriched GO terms are listed, and “ribosome biogenesis,” “ribosome” and “gene expression” are the top three most prominent GO terms. Pathway analysis was performed to determine the significantly affected pathways in parasites following tylosin treatment. As shown in Fig. 2f, pathways like “ribosome biogenesis in eukaryotes” and “ribosome” are remarkably affected.

### Anti-*Toxoplasma* activity of tylosin in vivo

**Fig. 3:**
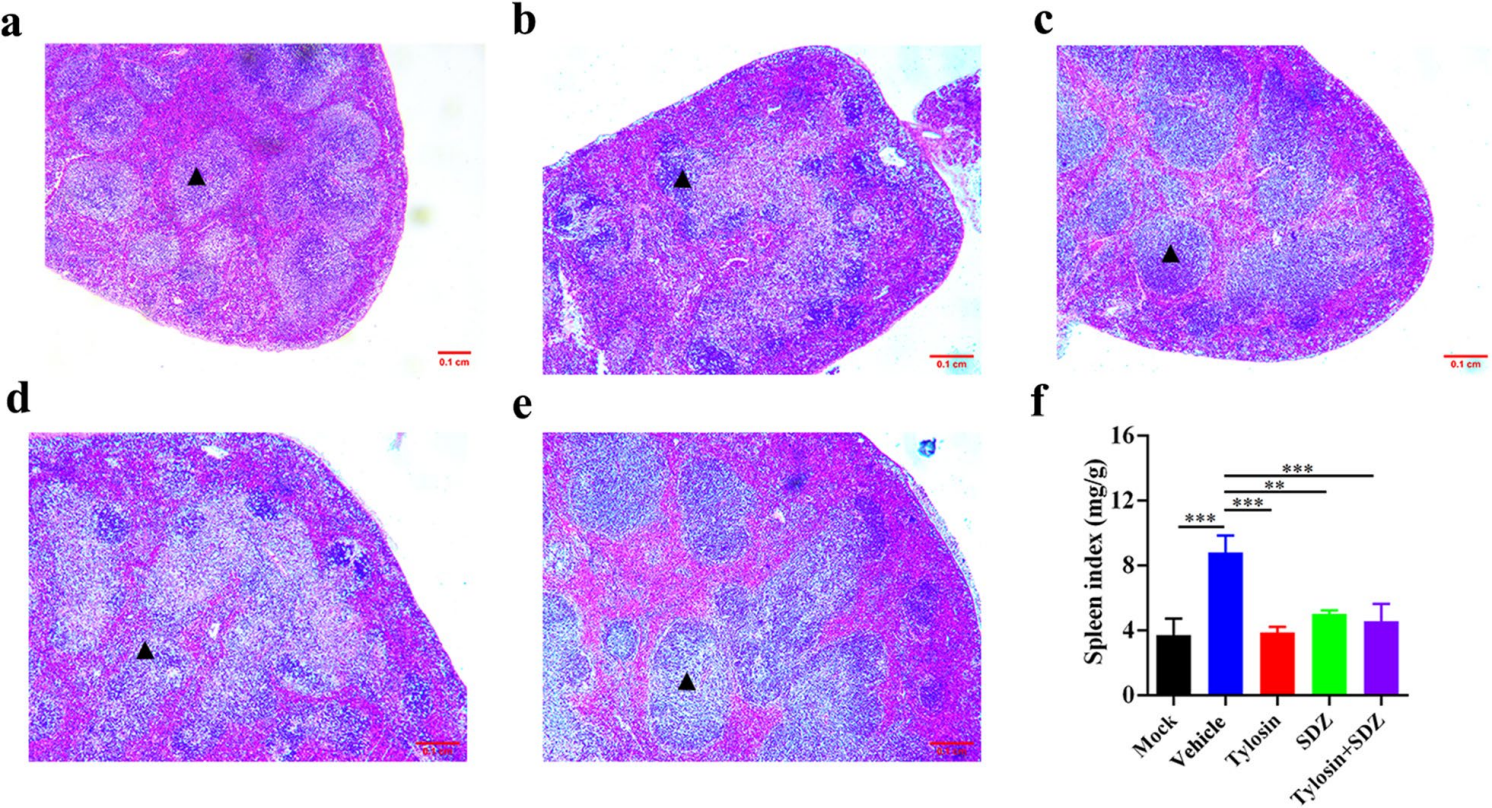
Effect of tylosin treatment on histopathological changes in mouse spleen infected with *Toxoplasma gondii* RH strain. **a** Healthy mice in mock group. Infected mice were administered vehicle (0.1% DMSO) (**b**), tylosin (**c**), SDZ (**d**) or tylosin + SDZ (**e**). Triangle indicates the white pulp. **f** Spleen index of mice. Values are means ± SD. The spleen index is expressed as the ratio (mg/g) of spleen wet weight/body weight. One-way ANOVA followed by Tukey’s multiple comparison is used to test differences between groups; **P < 0.01, ***P < 0.005

**Fig. 4:**
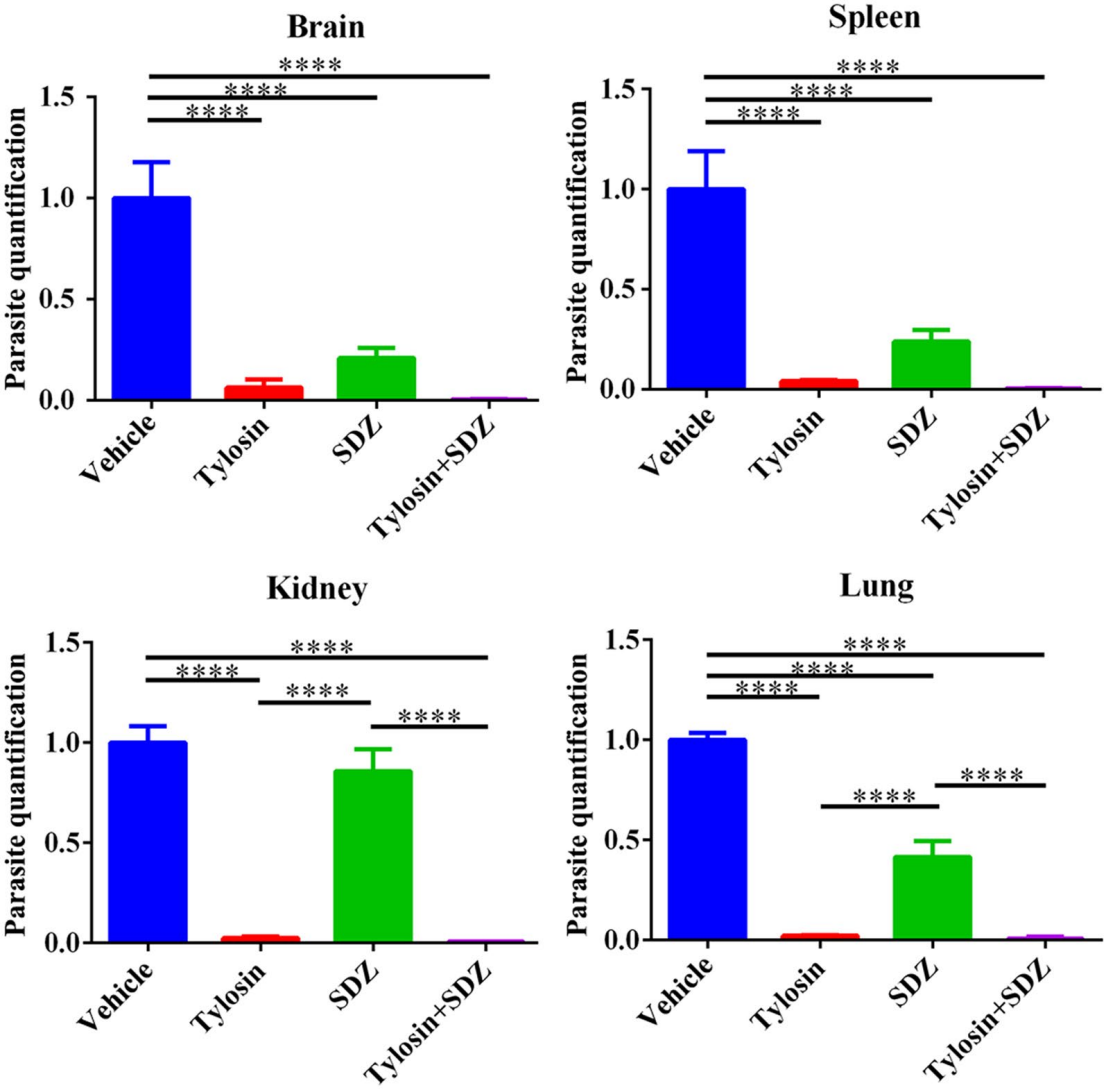
Effect of tylosin treatment on parasite burden in different tissues. **a** Brain. **b** Spleen. **c** Kidney. **d** Lung. One-way ANOVA followed by Tukey’s multiple comparison is used to test differences between groups; ****P < 0.001

**Fig. 5:**
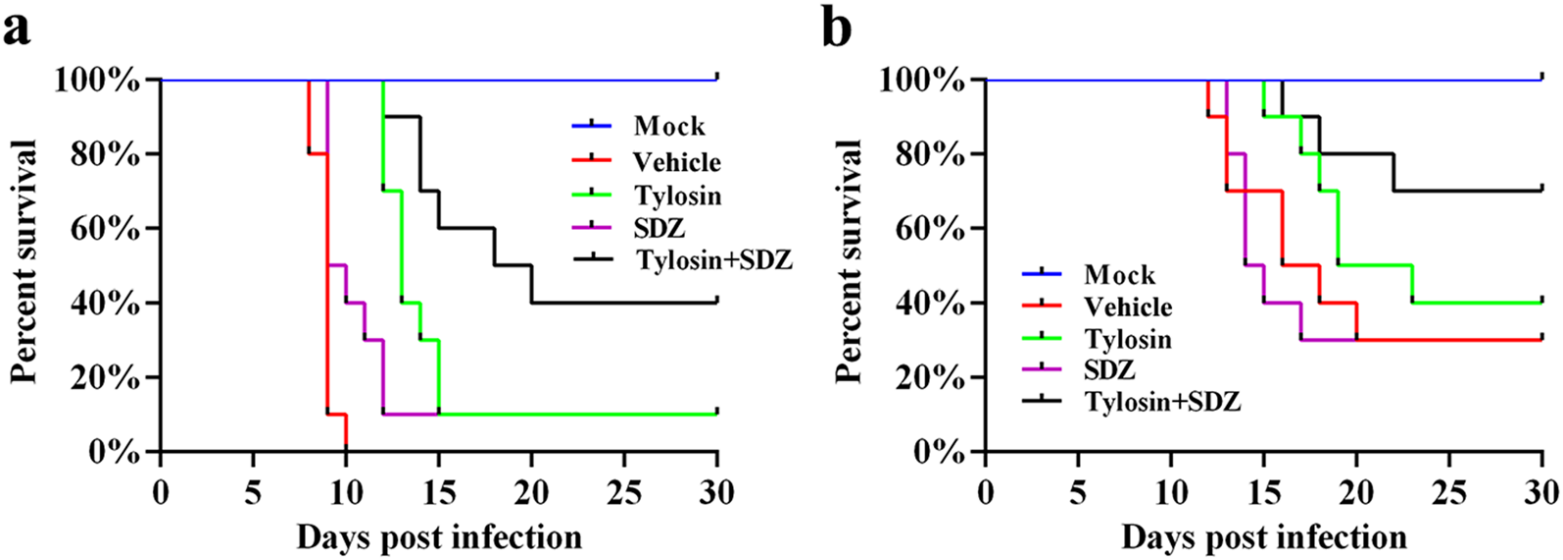
Effects of tylosin on virulent or avirulent *Toxoplasmos gondii* infections in KM mice. Female KM mice (*n* = 10) were intraperitoneally infected with *n* = 100 RH tachyzoites (**a**) or *n* = 200 ME49 tachyzoites (**b**). Meanwhile, a negative control group was mock infected with sterile PBS alone. Infected mice were then treated with intraperitoneal injection of tylosin (100 mg/kg/day), oral administration of sulfadiazine sodium (SDZ, 200 mg/kg/day), tylosin + SDZ, or vehicle. The survival of mice was monitored daily

To test the in vivo anti-*Toxoplasma* activity of tylosin, KM mice were infected intraperitoneally with 100 *T. gondii* RH tachyzoites or 200 ME49 tachyzoites. Mice in mock group were intraperitoneally injected with sterile PBS. Infected mice were administered vehicle, tylosin, sulfadiazine sodium (SDZ) or tylosin + SDZ for 7 consecutive days. At 7 days post infection, mice in the vehicle group showed signs of acute toxoplasmosis, including anorexia, weight loss, edema and messy hair. Compared with the mock group and treatment groups, mice in vehicle group developed severe splenomegaly. Using H&E staining, spleen samples were examined for histopathological damage. As shown in Fig. 3b, mice in the vehicle group exhibited a disruption of their splenic architecture with reduced white pulp and increase in the number of megakaryocytes. Meanwhile, the spleen index of mice treated with tylosin was significantly reduced compared with mice in the vehicle group (*P* < 0.005) (Fig. 3f). In addition, parasite burdens in different tissues (brain, spleen, kidney, and lung) were significantly decreased following drug treatments (Fig. 4). As shown in Fig. 5a, tylosin treatment prolonged survival time and resulted in 10% of mice surviving from *T. gondii* RH strain infection. The combination of tylosin and SDZ exhibited a better synergistic effect and increased the survival rate to 40%. As shown in Fig. 5b, tylosin treatment exhibited 40% survival from the ME49 infection. Mice treated with the combination of tylosin and SDZ exhibited 70% survival rate.

## Discussion

Toxoplasmosis ranks as one of the world's most common and neglected diseases caused by a protozoan parasite. Primary infection with *T. gondii* during pregnancy and recrudescence in immunocompromised individuals are two major clinical presentations. The exploration of novel antitoxoplasmic compounds will provide new therapeutic options for managing toxoplasmosis. In this study, we found that tylosin exhibited anti-*T. gondii* activity in vitro and in vivo. Additionally, the combination of tylosin and sulfadiazine sodium showed a better therapeutic effect.

During infection in the intermediate host, *T. gondii* undergoes stage conversion between the rapidly dividing tachyzoite that is responsible for acute toxoplasmosis and relatively non-immunogenic, dormant bradyzoite stage [20]. Previous studies showed severity of toxoplasmosis manifestation depends on the distribution and density of parasites [21, 22]. During acute infection, *T. gondii* tachyzoites disseminate throughout the body and cause multiple organ damage. The detection of parasite load has been used to assess toxoplasmosis severity and anti-parasite therapy effect, and it acts as an indicator for judging prognosis [23, 24]. In this study, we found that tylosin significantly inhibited parasite replication and decreased parasite burdens in multiple mouse organs. Splenomegaly is one of the most prominent clinical characteristics in mouse models of *T. gondii* infection [25]. Spleen index is considered the most reliable measurement for splenomegaly [26]. In this study, treatment with tylosin significantly reduced the spleen index in *T. gondii*-infected mice. Meanwhile, H&E staining revealed that splenic microarchitecture was substantially altered during acute *Toxoplasma* infections, which improved dramatically after tylosin treatment. These data indicate that tylosin can effectively relieve the splenomegaly caused by *T. gondii* infection.

Ribosomes are remarkably abundant in cells and serve as the site of protein synthesis. The ribosome is one of the best antibiotic targets. To date, more than half of approved antibiotics inhibit cell growth by interfering with ribosome function [27]. Macrolides are one of the most clinically important antibiotics; they inhibit bacterial protein synthesis by reversibly binding to the 50S unit of the ribosome. Macrolides are used to treat a wide range of infections caused by gram-positive and -negative bacteria. Some macrolides display pharmaceutical activity against intracellular pathogens. For example, balticolid, a 12-membered macrolide produced by a marine-derived fungus, has been shown to display anti-HSV activity [28]. Cyphomycin, caniferolide C and GT-35 are isolated from *Streptomyces* sp. and show potent antiprotozoal activity against intracellular leishmanial amastigotes [29]. Azithromycin and its derivatives exhibit excellent antimalarial activity by inhibiting parasite invasion and direct killing effects on asexual blood-stage parasites [30]. Macrolide antibiotics, such as spiramycin and azithromycin, exhibit inhibitory activity on the replication of intracellular *Toxoplasma* tachyzoites [31]. Tylosin is a macrolide antibiotic used as an antimicrobial growth promoter in animals. In this study, *T. gondii* exhibited a profoundly altered transcriptomic profile after tylosin treatment. Differential expression analysis identified 2401 DEGs (Fig. 2d). Through Gene Ontology analysis, “ribosome biogenesis (GO:0042254)” and “ribosome (GO:0005840)” are significantly enriched, which indicated an overall decrease in ribosomal activities. Our transcriptome data suggest that the ribosome is a particularly promising target for drug development against *T. gondii* infection.

## Conclusions

Tylosin significantly inhibited the proliferation of *T. gondii* in vitro and in vivo. Furthermore, administration of tylosin significantly extended the survival time and increased the survival rate of mice infected with *T. gondii* RH strain or ME49 strain. Importantly, the combined use of tylosin and SDZ significantly enhanced these effects compared with each of the monotherapies. Thus, our results suggest that tylosin may be a promising agent to treat toxoplasmosis in the future.

### Supplementary Information

**Additional file 1: Table S1.** DE mRNAs of *Toxoplasma gondii* after tylosin treatment.

## Data Availability

The high-throughput data are available in the NCBI database. The accession number for the mRNA-seq data reported in this paper is PRJNA951490.
